# The Dedicated Emergency Physician Model of emergency care is associated with reduced pre-hospital transportation time: A retrospective study with a nationwide database in Japan

**DOI:** 10.1371/journal.pone.0215231

**Published:** 2019-04-16

**Authors:** Hidenori Higashi, Reo Takaku, Atsushi Yamaoka, Alan Kawarai Lefor, Takashi Shiga

**Affiliations:** 1 Department of Emergency and Critical Care Medicine, Japanese Red Cross Wakayama Medical Center, Wakayama City, Wakayama, Japan; 2 Institute for Health Economics and Policy, Minato-ku, Tokyo, Japan; 3 Faculty of Economics of Kobe University, Nada-ku Kobe City, Hyogo, Japan; 4 Department of Surgery, Jichi Medical University, Shimotsuke, Tochigi, Japan; 5 Department of Emergency Medicine, International University of Health and Welfare, Minato-ku, Tokyo, Japan; University of Mississippi Medical Center, UNITED STATES

## Abstract

In Japan, the increasing number of patients needing emergency medical care due to population aging is a major public health problem. Recently, emergency medicine in Japan has seen a growth in the number of Dedicated Emergency Physician Model style departments. We aimed to determine whether there is an association between Dedicated Emergency Physician Model emergency care and pre-hospital transportation time. We conducted a secondary analysis of a Japanese national pre-hospital database from 2010 to 2014. Three regions (group 1: Urayasu city and Ichikawa city in Chiba prefecture, group 2: Kamakura city, Chigasaki city, Fujisawa city and Zushi city in Kanagawa prefecture, and group 3: Fukui city in Fukui prefecture) were evaluated as Dedicated Emergency Physician Model emergency medicine areas. We compared transportation times in these areas with all municipalities in the same prefectures, and with a nearby area using multivariate linear regression with cluster adjustment. The variables used for adjustment are the time from Emergency Medical Services activation to the scene, month, day of the month, day of the week, time of day, age, gender, type of injury, severity, and location of call. Compared with all municipalities in each prefecture there were significant reductions in pre-hospital transportation time: 4.2 minutes (95% confidence interval, 0.9 to 7.5, p<0.05) in Group 1, 6.2 minutes (95%CI, 2.9 to 9.6, p<0.01) fin Group 2 and 7.5 minutes (95%CI, 6.0 to 9.0, p<0.01) in Group 3. Compared with nearby areas, there were statistically significant reductions in transportation time in Group 1, 6.8 minutes (95%CI, 0.7 to 12.8, p<0.05) and in Group 2, 6.8 minutes (95%CI, 3.7 to 9.9, p<0.05). There was a trend for reduced transportation time in Group 3, 2.3 minutes, (5.3 to -0.6, p<0.1). Areas with a Dedicated Emergency Physician Model are associated with reduced pre-hospital transportation time.

## Introduction

An increasing number of emergency medical care patients due to population aging is a major public health problem in many nations [1–5]. In Japan, the number of ambulance transports per 10,000 population doubled and transportation time from Emergency Medical Services activation to hospital arrival increased about 1.5 times (24.4 minutes to 39.3 minutes) in the past two decades [6]. One of the reasons for transportation delay is an imbalance of supply and demand.

In Japan, the emergency care system is traditionally different from that in some other countries. In the majority of primary and secondary clinics and hospitals, emergency care is provided either by on-call physicians trained in any of a wide range of medical specialties or moonlighting physicians. These physicians often have no general skills for emergency care. In a few areas in Japan, care is provided according to a Dedicated Emergency Physician model and plays an important role to meet the increasing demand.

Multiple descriptive studies suggest that patient characteristics are related to ambulance acceptance or overuse of Emergency Medical Services [7–11]. An interventional study reported that information and communication technology shortened transportation time [12]. In contrast, the evidence for the effect of Dedicated Emergency Physician Model emergency care to reduce transportation time remains scarce.

To address the knowledge gap in the literature, we conducted a secondary analysis of a nationwide pre-hospital database to determine whether there is an association between Dedicated Emergency Physician Model care and reduction in transportation time.

## Methods

### Dedicated Emergency Physician Model emergency care in Japan

Currently, institutions in Japan adopt two different models of emergency medical care, the critical care model and the Dedicated Emergency Physician Model. The critical care model focuses on tertiary-level patients and is responsible for fewer than 5% of all emergency patients [13]. Traditional Japanese emergency physicians engage in the care of critically ill patients only. The majority of emergency care is provided either by on-call physicians trained in any specialty or moonlighting physicians. These physicians often have limited skill for emergency care. In this system, a junior resident or nurse sees the patient first and then assigns the patient to a particular department. Currently, in Japan, this is the most common model of practice. Problems arise when the patients are misdiagnosed and mis-assigned, or when a patient has multiple problems that involve several departments, as is often seen in patients with traumatic injuries.

The Dedicated Emergency Physician Model emergency care is a system where emergency physicians dedicate themselves to emergency patient care and they always work in shifts. This model of care is in widespread use in North America. Physicians take care of patients regardless of a patient’s condition or age. They are not involved in inpatient care and are involved in diagnosis, initial care, and advanced triage (disposition).

In Japan, the number of emergency physician is less than in the United States of America. The average number of emergency physicians is only 3,413 from 2010–2014 in Japan. The number of emergency physician per 10,000 populations is 1.24 in USA and 0.26 in Japan. In addition, Dedicated Emergency Physician Model physicians comprise less than 10% of the total emergency physician in Japan [14]. Therefore, a Dedicated Emergency Physician Model emergency medicine is provided in only limited areas.

### Emergency medical service system in Japan

When emergency patients call for Emergency Medical Services, on-scene Emergency Medical Services personnel determine the appropriate hospital in the area that is best able to treat the patients according to their symptoms and conditions. The Emergency Medical Services personnel then transport the patient to the selected hospital after obtaining the hospital staff’s agreement. Due to the emergency care models of care mentioned above and no laws controlling emergency care with negative sanctions such as the Emergency Medical Treatment and Active Labor Act (USA), difficulty in transporting the patient to an accepting hospital can occur at the scene in Japan. As a consequence, the transportation time from Emergency Medical Services activation to hospital arrival lengthens and delays the initiation of emergent treatment, which might lead to a worse patient outcome [6].

### Study design, population, and setting

This is a retrospective observational study based on a Japanese national database from 2010 to 2014 [15]. All emergency patients who called ambulances and were transported to hospitals were registered in our study. We selected three medical care areas (group 1: Urayasu city and Ichikawa city in Chiba prefecture, group 2: Kamakura city, Chigasaki city, Fujisawa city and Zushi city in Kanagawa prefecture, and group 3: Fukui city in Fukui prefecture) as Dedicated Emergency Physician Model emergency medicine areas. Each of these three areas has some Dedicated Emergency Physician Model emergency medicine hospitals (group 1: Tokyo Bay Urayasu Ichikawa medical center, group 2: Shonan Kamakura general hospital, group 3: Fukui prefectural hospital). These hospitals have Dedicated Emergency Physician Model emergency departments which do not refuse emergency patients and have more than 15 Dedicated Emergency Physician Model emergency physicians including senior staff and senior residents. For each Dedicated Emergency Physician Model area, we selected a nearby area, geographically next to the Dedicated Emergency Physician Model area and comparable in terms of population size, geographical size, and geographical location, for comparison. The Dedicated Emergency Physician Model areas were compared with all other municipalities in the same prefecture and with the nearby area. The nearby comparison area is Funabashi city for group 1, Odawara city, Isehara city, Hatano city and Sagamihara city for group 2, and Echizen city, Sabae city for group 3. Records with missing data were excluded from the analysis. Ambulance records are considered administrative records, and the requirement of obtaining patients’ informed consent was waived because the data are anonymous. This study was approved by the Ethics Committee of the Japan Red Cross Wakayama Medical Center (Approval Number: 570).

### Data collection and quality control

Data were uniformly collected using specific data collection forms and included age, gender, location of call, chronological factors such as time of day or day of week, time course of transport such as time of emergency call, time spent in contact with the patient, and time of hospital arrival, type of injury, and severity. The physicians who treat the patients subjectively evaluate their severity at the time of hospital arrival. EMS personnel complete a data form upon receiving a request for EMS transportation. Subsequently they record patient data obtained during transport. Upon arrival at the receiving hospital, a physician assessment is provided to the EMS team to complete the data form. Each data form is double checked by peer EMS personnel to ensure data accuracy. Finally, a designated supervising officer at each fire station assures the completeness of transport data. These data are an administrative record by the fire departments which do not require or connect to patient medical records.

### Outcome measures

The primary outcome measure is transportation time from arrival at the scene to arrival at the hospital in geographic areas using the Dedicated Emergency Physician Model for emergency care and control groups.

### Statistical analysis

Multivariate linear regression analysis was used to investigate the association between the Dedicated Emergency Physician Model of emergency care and a reduction in pre-hospital transportation time. The standard errors were clustered at the city level. As covariates, the time from Emergency Medical Services activation to the scene, month, day of the month, day in the week, time in the day, age, gender, type of injury, severity, and location of call were controlled for, based on a priori knowledge [7, 8]. To examine the multicollinearity of the models, we calculated a variance inflation factor for each model. We also divided the study population into two groups, high severity (includes patients classified as severe and dead) and low severity (includes patients classified as mild and moderate) and performed subpopulation analyses for each group. Data were analyzed using Stata version 14 (College Station, TX). All tests were two-tailed, and p values <0.05 were statistically significant.

## Results

**Fig 1:**
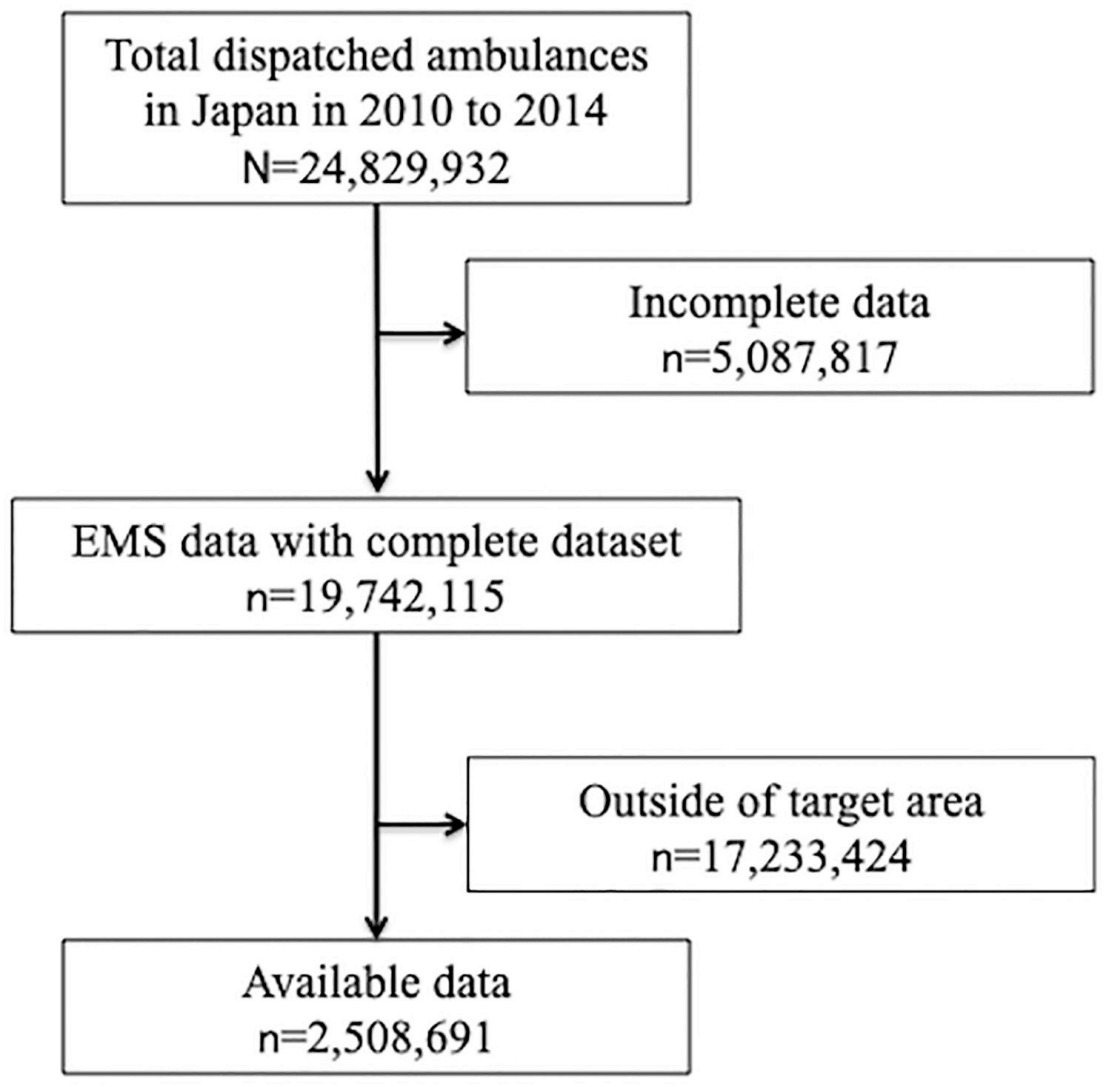
Patient flow. EMS, emergency medical services.

During the study period, a total of 24,829,932 emergency patients were documented in the national database. A total of 5,087,817 were excluded for missing data. In the target area of this study, a total of 2,508,691 were enrolled. Of these, the number of emergency patients in each area is 529,094 in Group 1, 1,864,321 in Group 2 and 115,276 in Group 3. A total of 645,869 patients were excluded for missing data in the target area. (Fig 1).

Baseline characteristics of study patients in each area are shown in Table 1. Medical illness is the most common reason for transportation and natural disasters is the least common in each area. More than half of all patients were judged to be mild to moderate severity in each area. More than half of the calls were from the patient’s home. About half of the calls are during nighttime.

**Table 1:** Patient characteristics.

	Chiba- Group 1	Kanagawa- Group 2	Fukui- Group 3
Observations	529,094	1,864,321	115,276
Age, median (IQR)	65 (39–79)	65 (39–80)	71 (47–82)
Female, n (%)	250,113 (47.3)	883,146 (47.4)	54,778 (47.5)
Time from emergency call to the scene, mean (min)	9	8	7
**Transport reason, n (%)**			
Fire accident	667 (0.1)	2,003 (0.1)	162 (0.1)
Natural disaster	65 (0.01)	345 (0.02)	18 (0.02)
Water accident	153 (0.03)	568 (0.03)	148 (0.1)
Traffic accident	53,527 (10.1)	166,798 (8.9)	15,071 (13.1)
Industrial accident	5,128 (1.0)	14,845 (0.8)	1,116 (1.0)
Disease and injury during sports	3,357 (0.6)	13,044 (0.7)	964 (0.8)
Other injury	75,446 (14.3)	291,332 (15.6)	16,461 (14.3)
Assault	3,713 (0.7)	13,449 (0.7)	348 (0.3)
Self-induced trauma	4,184 (0.8)	16,012 (0.9)	796 (0.7)
Medical illness	334,236 (63.2)	1,223,355 (65.6)	68,027 (59.0)
Transfer to a different hospital	46,577 (8.8)	119,943 (6.4)	12,106 (10.5)
**Severity, n (%)**^†^			
Death	6,892 (1.3)	23,112 (1.2)	2,115 (1.8)
Severe	38,474 (7.3)	157,300 (8.4)	17,654 (15.3)
Moderate	216,686 (41.0)	719,351 (38.6)	51,529 (44.7)
Mild	267,042 (50.5)	964,558 (51.7)	43,978 (38.2)
Location, n (%)			
Home	307,345 (58.1)	1,111,088 (59.6)	60,809 (52.8)
Public space	127,450 (24.1)	428,025 (23.0)	30,191 (26.2)
Workplace	12,519 (2.4)	41,772 (2.2)	2,780 (2.4)
Road	75,813 (14.3)	264,023 (14.2)	18,492 (16.0)
Others	5,967 (1.1)	19,413 (1.0)	3,004 (2.6)
Nighttime, n(%)^‡^	266,644 (50.4)	961,797 (51.6)	56,101 (48.7)

Abbreviation: IQR, interquartile range

^†^The treating physicians subjectively evaluate their severity at the time of hospital arrival

^‡^Total number of emergency call from 5 p.m. to 9 a.m.

Results of multivariate linear regression analysis and the effects of covariate factors are shown in Table 2.

**Table 2:** Results of multivariate linear regression analysis.

	Chiba- Group 1			Kanagawa- Group 2			Fukui- Group 3		
Comparison group	All other areas	Other cities	Nearby area	All other areas	Other cities	Nearby area	All other areas	Other cities	Nearby area
Observations	529,094	427,720	103,241	1,864,321	1,793,711	465,817	115,276	46,223	60,527
Difference in transportation time (min)^†^	-4.17**	-3.2	-2.34*	-6.25***	-6.06***	-6.76**	-7.54***	-11.26**	-6.78**
	[-7.48–-0.87]	[-7.42–1.03]	[-5.26–0.58]	[-9.60–-2.89]	[-9.58–-2.54]	[-12.79–-0.73]	[-9.03–-6.05]	[-18.51–-4.00]	[-9.87–-3.68]
Effect of time from emergency call to the scene^‡^	1.37***	1.38***	1.32***	1.39***	1.40***	1.43***	1.63***	1.52***	1.55***
	[1.24–1.49]	[1.25–1.51]	[1.17–1.46]	[1.23–1.55]	[1.23–1.58]	[1.28–1.57]	[1.42–1.85]	[1.25–1.79]	[1.20–1.87]
Adjusted R-squared	0.11	0.102	0.139	0.153	0.155	0.203	0.292	0.318	0.316
Transportation time, mean (min)	44.32	43.05	42.14	38.77	38.66	36.27	30.89	27.65	28.34

As covariates, the time from Emergency Medical Services activation to the scene, month, day of the month, day in the week, time in the day, age, gender, type of injury, severity, and location of call were controlled for

Robust standard errors clustered at levels of the fire station are reported in parenthesis

^†^Difference of transportation time subtracting transportation time of each control group from transportation time of target areas

^‡^The degree of extension of transportation time per minute increase in time from emergency call to the scene

*** p<0.01

** p<0.05

* p<0.1

Compared with all other areas, there were reductions in transportation time in the areas served by the Dedicated Emergency Physician Model: 4.2 minutes (95% confidence interval, 7.5 to 0.9, p<0.05) in Group 1, 6.2 minutes (95% CI, 2.9 to 9.6, p<0.01) in Group 2 and 7.5 minutes (95%CI, 6.0 to 9.0, p<0.01) in Group 3. When compared with nearby comparison areas, there were reductions in transportation time with statistical significance in Group 2 by 6.8 minutes (95%CI, 0.7 to 12.8, p<0.05) and in Group 3 by 6.8 minutes (95%CI, 3.7 to 9.9, p<0.05). There was a trend toward a reduction in transportation time in Group 1 of 2.3 minutes, (5.3 to -0.6, p<0.1). For each model, we calculated variance inflation factors. There is no multicollinearity because the variance inflation factor of each model is less than 10.

The results of subgroup analyses are shown in Table 3. In both the high and low severity groups, there were reductions in transportation time with statistical significance compared with all other areas.

**Table 3:** Results of subgroup analysis by severity.

	Chiba- Group 1			Kanagawa- Group 2			Fukui- Group 3		
	All other areas	Other cities	Nearby area	All other areas	Other cities	Nearby area	All other areas	Other cities	Nearby area
**Severe or dead**									
Observations	45,366	32,933	8,308	180,412	173,164	44,420	19,769	6,746	10,040
Difference in transportation time (min)^†^	-3.50**	-1.52	-4.41*	-2.86**	-2.52*	-4.66*	-10.31***	-15.20**	-7.83**
	[-6.57–-0.43]	[-4.81–1.76]	[-9.46–0.64]	[-5.46–-0.26]	[-5.14–0.10]	[-9.74–0.41]	[-12.87–-7.75]	[-27.21–-3.19]	[-15.11–-0.55]
Adjusted R-squared	0.111	0.106	0.143	0.126	0.13	0.16	0.26	0.337	0.295
Transportation time, mean (min)	45	42	44	38	38	37	33	29	29
**Mild or moderate**									
Observations	483,728	394,787	94,933	1,683,909	1,620,547	421,397	95,507	39,477	50,487
Difference in transportation time (min)^†^	-4.26**	-3.38	-2.15*	-6.59***	-6.42***	-6.96**	-7.12***	-10.28**	-6.57***
	[-7.71–-0.80]	[-7.82–1.07]	[-4.89–0.60]	[-10.05–-3.12]	[-10.06–-2.77]	[-13.10–-0.81]	[-8.60–-5.63]	[-16.95–-3.60]	[-8.86–-4.29]
Adjusted R-squared	0.114	0.106	0.144	0.16	0.162	0.213	0.318	0.323	0.328
Transportation time, mean (min)	44	43	42	39	39	36	30	27	28

As covariates, the time from Emergency Medical Services activation to the scene, month, day of the month, day in the week, time in the day, age, gender, type of injury, severity, and location of call were controlled for

Robust standard errors clustered at levels of the fire station are reported in parenthesis

^†^Difference of transportation time subtracting transportation time of each control group from transportation time of target areas

*** p<0.01

** p<0.05

* p<0.1

## Discussion

In this retrospective observational study of over 2,500,000 pre-hospital transports, we found an association between use of a Dedicated Emergency Physician Model of care with reduced transportation time. This observation suggests that the Dedicated Emergency Physician Model of emergency care plays an important role to respond to the increasing number of patients requiring transportation by ambulance.

A previous study demonstrated that pre-hospital factors such as being elderly, foreigners, loss of consciousness, holiday/weekend, nighttime, gas poisoning, trauma by assault, self-induced drug/gas abuse poisoning, and self-induced trauma were positively associated with difficulty in hospital acceptance [7]. Another study showed that patients with traumatic injuries, pediatric patients, male gender, moderate to severe grade trauma, holidays and weekends and nighttime were positively associated with difficulty in hospital acceptance [8]. These studies examined patient characteristics. The present study is unique it focuses on the difference the system of care, especially the effect of having a Dedicated Emergency Physician Model of emergency care.

The effect of the Dedicated Emergency Physician Model to result in reduced transportation time, could be explained by strengths of emergency medicine practiced according to the Dedicated Emergency Physician Model. Each hospital which uses this model of care has a training program to teach this model of emergency medicine and physicians can manage emergency patients regardless of their condition or severity and can concentrate on initial emergency care. In addition, they can work with fewer burdens because of the shift work system. They have a greater capacity to accept multiple emergency patients. We suggest that these factors contribute to the observed reduction in transportation time.

This study has important implications for emergency medical systems. Previously, whether the Dedicated Emergency Physician Model of emergency care affects transportation time had not been emphasized. If this model becomes more accepted, transportation time will be reduced in many regions and it may contribute to improved prognosis. In addition, the Dedicated Emergency Physician Model might facilitate better medical care in communities with limited medical resources.

The present study has some limitations. First, the outcome measure of this study is the average pre-hospital transportation time. Due to this, the results do not reflect in-hospital outcomes. In-hospital data would be needed in a future study. Second, we need to be specific regarding the definition of the Dedicated Emergency Physician Model. In this study, we strictly defined the Dedicated Emergency Physician Model as a department which does not refuse emergency patients and has more than 15 emergency physicians. However, smaller hospitals in control groups may have mixed models of care such as a daytime Dedicated Emergency Physician Model and a nighttime traditional care model. Third, pre-hospital transportation time is partially dependent on the geographic distance from fire-station to the scene or from the scene to the hospital. We tried to control this factor by defining pre-hospital transportation time as from the scene to the hospital as well as adjusting for the time from the emergency call to the scene as one of the covariates. Fourth, these results may not be generalized because the present study is based on the Japanese national pre-hospital database and Japanese Emergency Medical Services systems and ER systems are different from other countries. Fifth, because of the nature of an observational study, we need to consider the possibility of unknown confounding factors that influenced our results. Sixth, we checked the number of physicians per 1000 person in 2010 (Japan Medical Association Research Institute 2013) since transportation time may be shorter in the areas with more physicians, even in the absence of a Dedicated Emergency Physician Model emergency system. However, we did not find large differences in all sites, though the number of physicians in Fukui city is twice as large as the optimum comparison group (i.e. Sabae and Echizen city).

## Conclusion

In this retrospective study using a large national database, areas with hospitals that employ a Dedicated Emergency Physician Model of emergency care are associated with reduced pre-hospital transportation time.
